# Identification and Localization of Breast Tumor Components via a Convolutional Neural Network Based on High-Frequency Ultrasound Combined With Histopathologic Registration: Prospective Study

**DOI:** 10.2196/81181

**Published:** 2026-01-23

**Authors:** Jia-Qian Yao, Wen-Wen Zhou, Zhi-Fei Chai, Fei Ren, Tong-Yi Huang, Tian-Tian Zhen, Hui-Juan Shi, Xiao-Yan Xie, Ze Zhao, Ming Xu

**Affiliations:** 1 Department of Medical Ultrasonics, The First Affiliated Hospital, Sun Yat-sen University, 58 Zhongshan 2nd Road, Guangzhou, 510080, China, +86-020-8776 518; 2 Department of Medical Ultrasonics, Suzhou Municipal Hospital Affiliated with Nanjing Medical University, Suzhou, China; 3 Institute of Computing Technology, Chinese Academy of Sciences, Beijing, China; 4 Department of Pathology, The First Affiliated Hospital, Sun Yat-sen University, Guangzhou, China

**Keywords:** breast, neoplasms, biopsy, ultrasonography, registration

## Abstract

**Background:**

Given the highly heterogeneous biology of breast cancer, a more effective noninvasive diagnostic tool that unravels microscopic histopathology patterns is urgently needed.

**Objective:**

This study aims to identify cancerous regions in ultrasound images of breast cancer via convolutional neural network based on registered grayscale ultrasound images and readily accessible biopsy whole slide images (WSIs).

**Methods:**

This single-center study prospectively included participants undergoing ultrasound-guided core needle biopsy procedures for Breast Imaging Reporting and Data System category 4 or 5 breast lesions for whom breast cancer was pathologically confirmed from July 2022 to February 2023 consecutively. The basic information, ultrasound image data, biopsy tissue specimens, and corresponding WSIs were collected. After core needle biopsy procedures, the stained breast tissue specimens were sliced and coregistered with an ultrasound image of a needle tract. Convolutional neural network models for identifying breast cancer cells in ultrasound images were developed using FCN-101 and DeepLabV3 networks. The image-level predictive performance was evaluated and compared quantitatively by pixel accuracy, Dice similarity coefficient, and recall. Pixel-level classification was illustrated through confusion matrices. The cancerous region in the testing dataset was further visualized in ultrasound images. Potential clinical applications were qualitatively assessed by comparing the automatic segmentation results and the actual pathological tissue distributions.

**Results:**

A total of 105 participants with 386 ultrasound images of breast cancer were included, with 270 (70%), 78 (20.2%), and 38 (9.8%) images in the training, validation, and test datasets, respectively. Both models performed well in predicting the cancerous regions in the biopsy area, whereas the FCN-101 model was superior to the DeepLabV3 model in terms of pixel accuracy (86.91% vs 69.55%; *P*=.002) and Dice similarity coefficient (77.47% vs 69.90%; *P*<.001). The two models yielded recall values of 54.64% and 58.46%, with no significant difference between them (*P*=.80). Furthermore, the FCN-101 model had an advantage in predicting cancerous regions, while the DeepLabV3 model achieved more accurate predictive pixels in normal tissue (both *P*<.05). Visualization of cancerous regions on grayscale ultrasound images demonstrated high consistency with those identified on WSIs.

**Conclusions:**

The technique for spatial registration of breast WSIs and ultrasound images of a needle tract was established. Breast cancer regions were accurately identified and localized on a pixel level in high-frequency ultrasound images via an advanced convolutional neural network with histopathologic WSI as the reference standard.

## Introduction

Breast cancer heterogeneity has induced challenges in treatment planning and follow-up management, which leads to unfavorable outcomes [1,2]. Currently, ultrasound is a widely used diagnostic tool for breast cancer management, particularly valuable in screening, positive diagnosis, and treatment response assessment [3]. However, the biological heterogeneity of breast cancers leads to varied morphological features on ultrasound [4], often resulting in malignancy underestimation and overestimation. Furthermore, there is a heterogeneous response to treatment among patients with breast cancer [5-7]. Curative effect assessment secondary to preoperative neoadjuvant treatment is largely based on cancer volume changes [8], as well as biopsy for further validation [9]. A more accurate noninvasive diagnostic tool that indicates living cancer cells in breast cancer is urgently needed [10-12].

Hematoxylin and eosin (H&E) staining of breast tissue captured via core needle biopsy (CNB) has been introduced to reflect the underlying cellular and molecular information [13-15]. Preoperative diagnosis and curative effect assessment of breast cancer can be undermined by insufficient and nonrepresentative tissue owing to the heterogeneous distribution of breast cancer [16]. Likewise, the partial samples obtained by CNB may not represent the entire lesion [17]. There remains a need for standardized methods or imaging biomarkers available for accurately localizing histopathological cancerous subregions.

The convolutional neural network (CNN), a developed type of deep learning algorithm, has shown remarkable performance in correlating macroscopic imaging and microscopic histopathologic microstructure. A previous study showed that a multimodal radiomics model combining ultrasound and whole slide image (WSI) can effectively distinguish between luminal and nonluminal breast cancers [18]. Other studies have explored using the deep learning algorithm for correlation between magnetic resonance imaging (MRI) and whole-mount specimen images to localize prostate cancer [19]. Theoretically, these approaches may also be applicable in the ultrasound identification and localization of cancerous regions in breast cancer. Nonetheless, few studies have focused on this aspect. The correlation of ultrasound modality and readily accessible biopsy WSI remains open to question.

Therefore, this study aims to identify and localize cancerous regions in breast cancer based on a CNN algorithm that integrates high-frequency ultrasound (HFUS) images with WSI histopathology. The predictive performance of the model will be assessed.

## Methods

### Study Population

Consenting participants were recruited between July 2022 and February 2023 and were included as the training, validation, and test population. The eligibility criteria included the following: (1) index lesion was defined as category 4 or 5 according to the fifth edition of the American College of Radiology Breast Imaging Reporting and Data System (BI-RADS) of ultrasound [20]; (2) index lesion was visible on HFUS, and complete imaging data were stored; (3) underwent ultrasound-guided breast lesion biopsy and the histopathology indicated breast cancer. Participants were excluded if (1) the biopsy specimen was incomplete or inaccessible; (2) the breast ultrasound images were incomplete; (3) there was a history of treatment for breast cancer (surgery, antihormonal therapy, immunotherapy, and radiation therapy); (4) the pathologic diagnosis was incomplete. The study sample included 163 consecutive participants undergoing ultrasound-guided breast biopsy for suspicion of cancer. Of these, three participants whose ultrasound images had poor quality and one who did not provide research consent were excluded. Participants with incomplete, fractional tissue specimens (n=2) and benign histology reports (n=52) were also excluded.

### Ultrasound and CNB Examination

**Figure 1.**
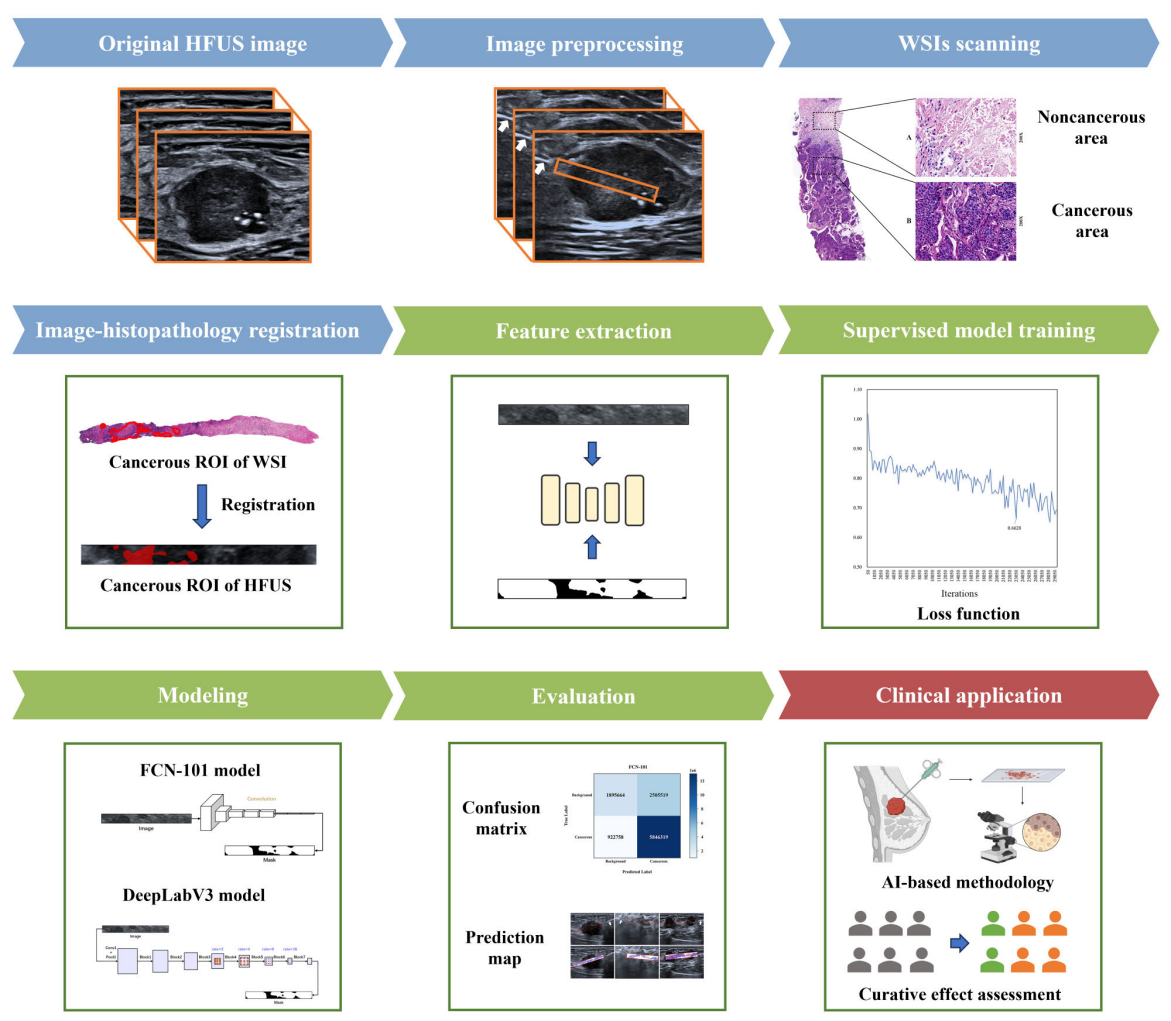
Workflow of the proposed cancerous regions identification protocol in this study. First, we got cropped HFUS images and corresponding spatially aligned biopsy WSIs. Second, a registration process was applied to achieve anatomic correlation. Third, the segmentation model was constructed using the FCN-101 and DeepLabV3 architectures. AI: artificial intelligence; HFUS: high-frequency ultrasound; ROI: region of interest; WSI: whole slide image.

The overall design of this study is shown in Figure 1. Expert radiologists with at least 10 years of experience performed breast ultrasound examinations and ultrasound-guided CNB procedures following the standard practice protocol. Location, number, and morphologic characteristics (size, shape, orientation, margin, echo pattern, vascularity, and calcifications) of lesions were identified and categorizations were assigned by the expert radiologists according to the fifth edition of ultrasound BI-RADS [20]. An Aplio i900 Ultrasound System (Canon) with an i24LX8 high-frequency linear probe (frequency range: 8.0‐18.0 MHz) was used to generate breast ultrasound images. All images were stored in DICOM format for subsequent analysis.

### WSI Acquisition

When breast cancer was suspected, an ultrasound-guided CNB procedure was performed by expert radiologists to determine the diagnosis. MAGNUM biopsy instruments (BARD) with disposable core tissue biopsy needles (16G, 22 mm, MN1620, BARD) were adopted. During the procedure, the needle tip was positioned approximately 0.5 cm from the target biopsy region. The radiologists aligned the ultrasound probe and needle to visualize the entire needle tract. Subtle needle deflections were occasionally observed. These deflections were dynamically corrected in real time by adjusting the needle trajectory. The radiologists captured two B-mode HFUS images per biopsy for subsequent registration, including one pre-fire and one post-fire. To ensure the specimens were representative, biopsies were taken by the radiologist from different regions of the lesion, typically 4‐6 samples, focusing on solid areas on B-mode ultrasound or areas with abundant vascularity on color Doppler flow imaging. When multiple lesions were encountered, the most suspicious lesion for malignancy was chosen for analysis. To facilitate the follow-up spatial registration of ultrasound and histopathologic images, the needle tip side of the biopsy specimen from the index lesion was stained with biological tissue dye (BIOGNOST), depicted in Figure S1 in Multimedia Appendix 1. After 2 to 5 minutes of coloration, the biopsy specimens were placed in 10% neutral buffered formalin for fixation and sent for histopathologic examination.

The tissue samples were fixed and oriented during the embedding process to preserve the longitudinal axis. Each 3 μm histological slide was then sectioned parallel to the initial needle trajectory, thereby ensuring that the analyzed WSI represented the same gross anatomical plane captured by HFUS. The tissue strips were very small, thus the dimensional changes and distortion introduced by histological processing were assumed to be limited. Each section was stained with H&E. For each participant, all slides stained with H&E were reviewed by two experienced breast pathologists (with 5 y and 10 y of experience), and the histopathologic type was reported. Scanning of the H&E slides was performed using a KFBIO Digital Pathology Slide Scanner (KF-PRO-020) with a 200X objective lens. Representative heterogeneous cancer cell distribution of breast cancer in biopsy WSI is depicted in Figure S2 in Multimedia Appendix 1.

### Imaging Registration

**Figure 2.**
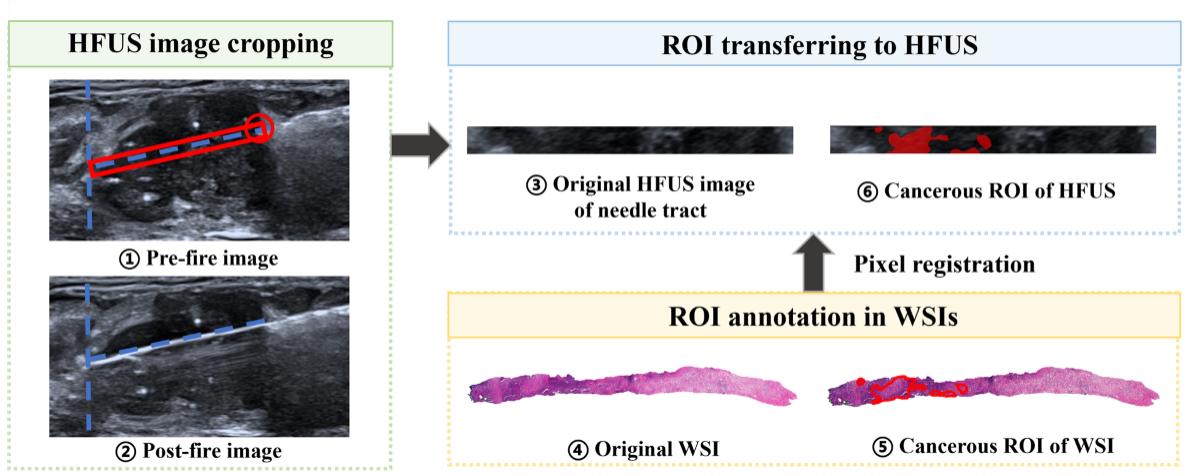
Spatial registration and mapping protocol between HFUS and WSI. We compared the pre-fire image and post-fire image to crop the needle tract in HFUS images. An experienced pathologist manually outlined the cancerous cells on WSI images using QuPath software (version 0.4.0 [21]), which were transferred to the cropped HFUS images, creating the labeled dataset for training. HFUS: high-frequency ultrasound; ROI: region of interest; WSI: whole slide image.

The expert pathologist with 10 years of experience used the open-source software QuPath (version 0.4.0) for digital histopathology analysis. The maximal cross-section of the extracted core was used for analysis. Cancer cells were identified based on nuclear atypia and mitotic figures. The regions of interest (ROIs) of cancerous regions were manually outlined on all high-resolution WSI slices, generating a per-pixel cancer cell labeling (Figure 2).

For 4‐6 samples from one lesion, the expert pathologist selected 1‐4 specimens for further registration processing. The method for selecting was based on three criteria: (1) intact and well-formed; (2) length matches the needle notch (22 mm); (3) clear and distinguishable staining. For each selected tissue sample, one radiologist with at least 5 years of experience correlated the WSI to two captured B-mode HFUS images. To ensure the spatial registration from WSI to the HFUS images, the radiologist compares the pre-fire and post-fire HFUS images using Photoshop software (version CC 2019; Adobe Inc). In the software, the needle tip position in the pre-fire image was taken as the starting point; actual length of the biopsy specimen was determined by the needle projection distance in the post-fire image. Then, the radiologist cropped the biopsy area in the pre-fire image for subsequent annotation and analysis. The detailed image cropping process is demonstrated in Figure 2. The HFUS image of the needle tract was then saved in JPG format. The ROIs outlined in WSIs were converted to correlated HFUS images via Labelme software, an open-source image annotation tool (version 5.1.0).

### Data Preprocessing

The HFUS images and their ROIs annotation were obtained to establish the CNN prediction model. Open-source libraries including Python (version 3.8.11; Python Software Foundation), Imgviz (version 1.5.1), and Numpy (version 1.21.6) were used to convert the dataset into the standard VOC format. To enhance the robustness and generalization capability of the model, data augmentation strategies, such as random horizontal flipping, rotation, and pixel transformations, were used to increase the generalizability of the model. The dataset was randomly divided into training (n=270, 70%), validation (n=78, 20.2%), and test (n=38, 9.8%) subsets using a 7:2:1 ratio with no overlap between the subsets. Image pixel values were normalized using the *z*-score method to reduce computation burden and accelerate model convergence.

### Model Development

The fully automated segmentation CNN model was designed to identify cancerous regions in breast cancer based on HFUS images, with the ROIs in WSI as the reference standard. The independent CNN model was trained with advanced FCN-101 [22] and DeepLabV3 [23] networks as the backbone, separately. During training, the models were adapted using the AdamW optimized algorithm and CosineAnnealing learning rate adjustment curve to improve efficiency. The weighted Dice loss function was used to mitigate the data imbalance during the training process, as plotted in Figure S3 in Multimedia Appendix 1. The iteration parameters with the lowest loss values were chosen, which were 0.6626 for the FCN-101 model and 0.7285 for the DeepLabV3 model. In the fine-tuned process, parameters were selected to construct the segmentation model when the best performance on the test dataset was achieved. Finally, cancerous regions in breast cancer were localized. More details on the CNN model are provided in Multimedia Appendix 1. The source code for our CNN models is available on GitHub [24].

### Statistical Analysis

All statistical analysis was conducted with IBM SPSS Statistics 25.0 (version 25.0; IBM Corp), Python (version 3.8.11; Python Software Foundation), and R software (version 4.2.1, R Foundation for Statistical Computing). Continuous variables were exhibited as means (SDs) or medians and IQRs and compared using a 1-sample *t* test or Mann-Whitney *U* test where appropriate. Categorical variables were expressed as counts and percentages and compared through *χ*^2^ tests. To assess and compare the segmentation performance of CNN models based on independent networks, pixel accuracy (PA), Dice similarity coefficient (DSC), mean Intersection over Union, precision, and recall were calculated. These metrics were computed separately for each test image, and the final performance values were obtained by averaging the image-level results across the entire dataset. To avoid inflated degrees of freedom arising from multiple images per participant, a paired-sample *t* test was performed at the participant level to compare the performance of FCN-101 and DeepLabV3 models. Normality of the paired differences was confirmed using the Shapiro-Wilk test. Detailed evaluation metrics were demonstrated in the Supplementary Methods in Multimedia Appendix 1. The confusion matrices were adopted to show the pixel-level classification of cancerous regions and normal tissue based on aggregated raw pixel counts. Two-tailed *P*<.05 was considered statistically significant.

### Ethical Considerations

This single-center prospective study involving human participants was approved by the local institutional review board of the First Affiliated Hospital of Sun Yat-sen University (Ethics [2023]842). Written informed consent was obtained from all participants prior to their inclusion in the study. This study was conducted in accordance with the Declaration of Helsinki and its subsequent amendments. All data were deidentified before analysis to ensure participant privacy. Participants did not receive any financial or material compensation, as all procedures were part of routine clinical care, and the analytical use of the resulting data was clearly explained to participants during enrollment. No identifiable images or personal information are included.

## Results

### Participant Characteristics

**Figure 3.**
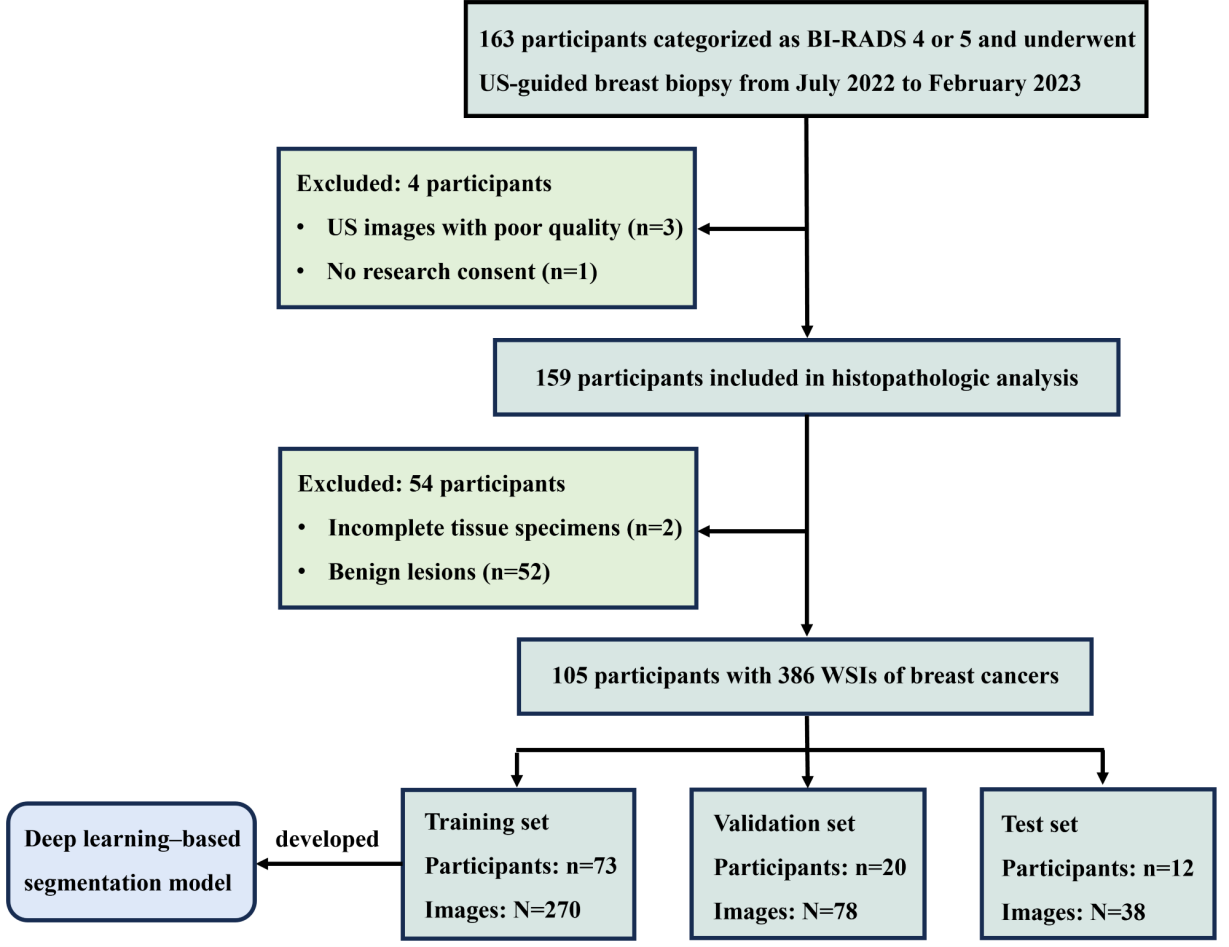
Flowchart showing inclusion and exclusion criteria of participants. BI-RADS: Breast Imaging Reporting and Data System; US: ultrasound; WSI: whole slide image.

A total of 163 individual participants received ultrasound-guided breast biopsy, and 58 participants were excluded (Figure 3). The final prospective dataset contained 105 participants (mean age, 53.7, SD 11.3 y old; all female) diagnosed as breast cancer with 386 HFUS images of the needle tract. All biopsies ultimately achieved successful targeting and adequate sampling in the 105 participants. Among them, 64 (87.7%), 19 (95%), and 9 (75%) invasive breast cancer and 9 (12.3%), 1 (5%), and 3 (25%) ductal carcinomas in situ were identified in the training, validation, and test subsets, respectively. Characteristics were compared between three subsets in Table 1 and no evidence of a statistical difference was observed, except for vascularity, where internal vascularity of breast lesions was observed in the training dataset (*P*=.03).

**Table 1.** Participants and breast cancer characteristics. Unless otherwise indicated, data are numbers of lesions, with the percentages in parentheses.

Characteristic	Training set (n=73)	Validation set (n=20)	Testing set (n=12)	*P* value
Age (y), mean (SD)	52.7 (SD 11.2)	57.8 (SD 13.2)	52.8 (SD 7.4)	.21
Lesion number, n (%)				.08
Solitary	64 (87.7)	19 (95)	8 (66.7)	
Numerous	9 (12.3)	1 (5)	4 (33.3)	
BI-RADS^a^ categorization, n (%)				.96
BI-RADS 4a	5 (6.8)	1 (5)	1 (8.3)	
BI-RADS 4b	9 (12.3)	3 (15)	1 (8.3)	
BI-RADS 4c	18 (24.7)	3 (15)	2 (16.7)	
BI-RADS 5	40 (54.8)	13 (65)	8 (66.7)	
Lesion location, n (%)				.68
Left breast	32 (43.8)	11 (55)	6 (50)	
Right breast	41 (56.2)	9 (45)	6 (50)	
Quadrant, n (%)				.30
Upper outer quadrant	45 (61.6)	9 (45)	5 (41.7)	
Lower outer quadrant	10 (13.7)	1 (5)	2 (16.7)	
Upper inner quadrant	12 (16.4)	6 (30)	3 (25)	
Lower inner quadrant	6 (8.2)	4 (20)	2 (16.7)	
Volume (mm^3^), mean (SD)	4.1 (SD 5.1)	3.4 (SD 2.8)	4.3 (SD 7.0)	.82
Orientation, n (%)				.22
Parallel	82 (89.1)	15 (75)	11 (91.7)	
Not parallel	10 (10.9)	5 (25)	1 (8.3)	
Echo pattern, n (%)				.78
Hypoechoic	86 (93.5)	20 (100)	12 (100)	
Heterogeneous	6 (6.5)	0 (0)	0 (0)	
Margin, n (%)				.52
Circumscribed	6 (8.2)	2 (10)	2 (16.7)	
Not circumscribed	67 (91.8)	18 (90)	10 (83.3)	
Shape, n (%)				.47
Oval/Round	3 (4.1)	0 (0.0)	1 (8.3)	
Irregular	70 (95.9)	20 (100.0)	11 (91.7)	
Calcification, n (%)				.40
Absence	36 (49.3)	25 (59.5)	8 (66.7)	
Calcification in a mass	37 (50.7)	17 (40.5)	4 (33.3)	
Vascularity, n (%)				.03
Absent	11 (15.1)	7 (35)	2 (16.7)	
Internal vascularity	56 (76.7)	11 (55)	7 (58.3)	
Vessels in rim	6 (18.2)	2 (10)	4 (33.3)	
Histopathologic pattern, n (%)				.13
Invasive breast cancer	64 (87.7)	19 (95)	9 (75)	
Ductal carcinoma in situ	9 (12.3)	1 (5)	3 (25)	

a BI-RADS: Breast Imaging Reporting and Data System.

### Model Evaluation

The segmentation capacity of the image-based CNN model based on FCN-101 and DeepLabV3 in the test dataset is provided in Table 2. The table presents image-level descriptive statistics, while all inferential comparisons were conducted at the participant level (n=12). In the test dataset, FCN-101 showed higher accuracy (PA: 86.91% vs 69.55%, *P*=.002), similarity (DSC: 77.47% vs 69.90%, *P*<.001), mean Intersection over Union (67.47% vs 60.29%, *P*<.001), and precision (66.01% vs 56.15%, *P*<.001) compared to DeepLabV3. There was no evidence of a difference in recall (54.64% vs 58.46%, *P*=.80) between the two algorithms.

**Table 2.** Comparison of prediction performance of FCN-101 and DeepLabV3 models in predicting cancerous regions in breast cancer in the test dataset. All metrics reflect image-level performance and are expressed as percentages. Statistical comparisons (*P* values) were performed at the participant level using paired analyses.

Evaluation metric	FCN-101, % (95% CI)	DeepLabV3, % (95% CI)	*P* value
PA^a^	86.91 (80.30-94.77)	69.55 (65.82-73.99)	.002
DSC^b^	77.47 (70.74-85.88)	69.90 (63.43-75.02)	<.001
mIoU^c^	67.47 (59.70-75.54)	60.29 (54.19-66.57)	<.001
Precision	66.01 (55.69-73.45)	56.15 (48.12-64.17)	<.001
Recall	54.64 (45.57-63.75)	58.46 (49.03-65.66)	.80

a PA: pixel accuracy.

b DSC: Dice similarity coefficient.

c mIoU: mean Intersection over Union.

**Figure 4.**
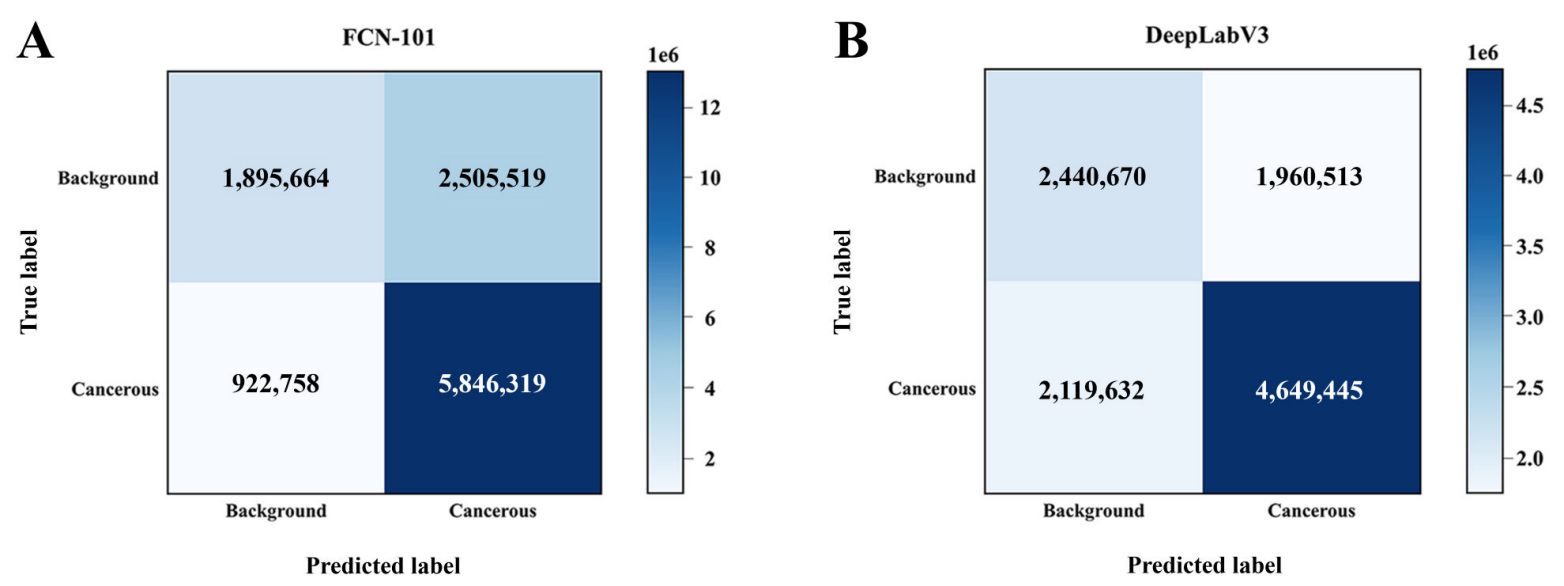
Confusion matrices for predicting cancerous regions. Confusion matrices were applied to summarize pixel-level classification outcomes aggregated across the entire test dataset for (A) FCN-101 and (B) DeepLabV3, indicating discordance or concordance with the ground truth (cancerous cell/background) from WSI results. WSI: whole slide image.

The confusion matrix presented in Figure 4 evaluates the pixel categorization capacity, providing further insight into the per-pixel predictions of each model. The results indicated that the FCN-101 model successfully predicted the majority of cancerous pixels (5,846,319 vs 4,649,445 pixels; *P*<.05). However, the DeepLabV3 model demonstrated more accurate predictive pixels in background components (2,440,670 vs 1,895,664 pixels; *P*<.05). Based on the pixel-wise confusion matrices, the recall and specificity of FCN-101 were 86.37% and 43.07%, while those of DeepLabV3 reached 68.69% and 55.46%.

### Model Visualization

**Figure 5.**
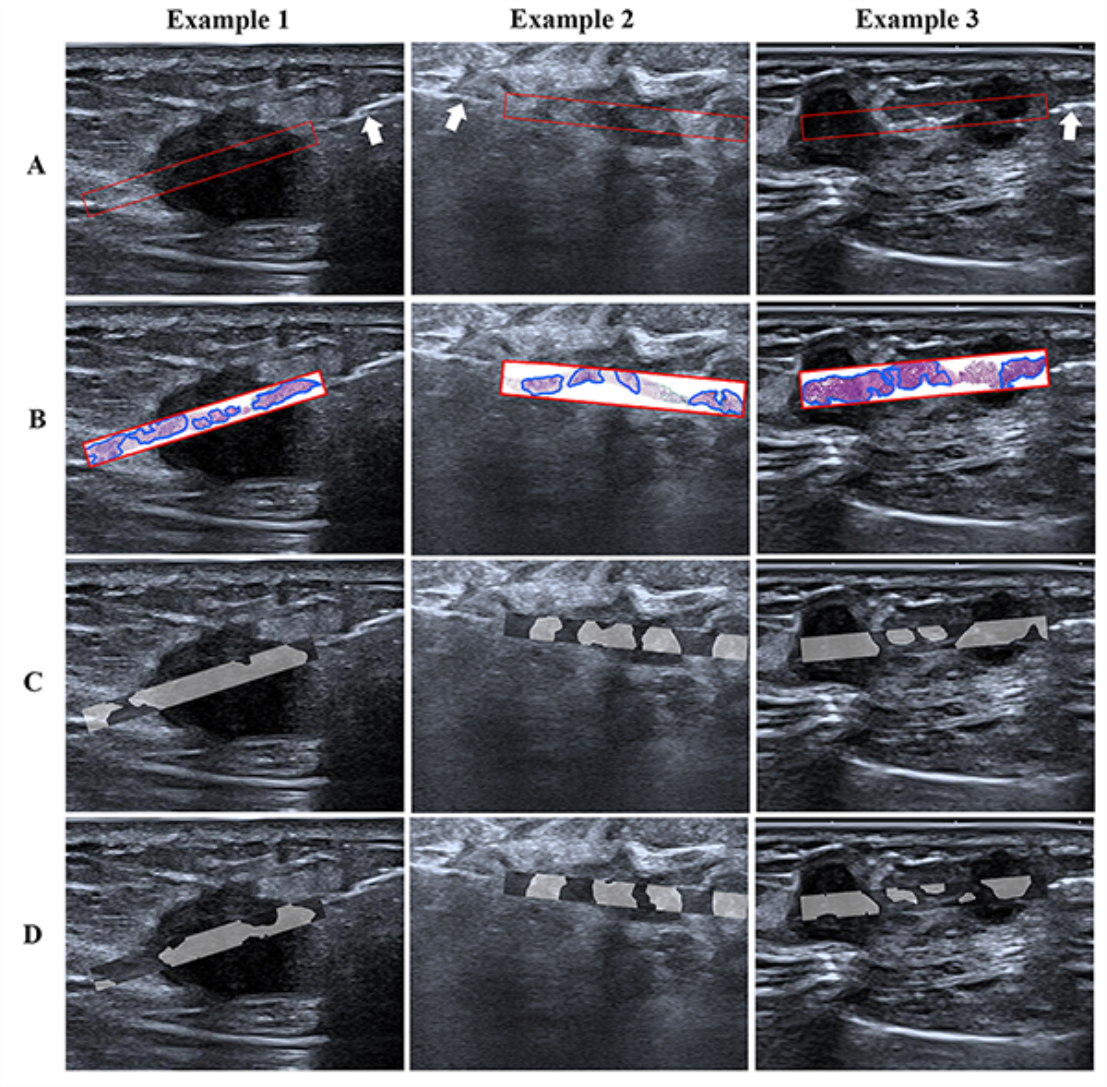
Examples of identifying cancerous regions in three participants with invasive breast cancer. (**A**) Original HFUS images, with biopsy needle (white arrow) pointing to the needle tract area (red frame). (**B**) Annotation of ground truth labels by an expert pathologist. (**C**) Prediction results of the FCN-101 model (gray area). (**D**) Prediction results of the DeepLabV3 model (gray area). HFUS: high-frequency ultrasound.

In the test dataset, the automatic segmentation results of cancerous regions based on the two networks were visually displayed and qualitatively compared with the gold standard to evaluate the predictive performance of the models. Cancerous regions in the CNB biopsy area of three different participants’ breast HFUS images were identified and localized in Figure 5, as generated by the FCN-101 and DeepLabV3 models. Cancerous region predictions by the two CNN models align closely with the actual histopathology. Additionally, even in challenging cases with unclear boundaries and mixed internal echoes, as represented in Example 2, both models were able to accurately predict the specific distribution of cancerous regions within the lesions.

## Discussion

### Principal Results

Breast cancer is a group of highly heterogeneous diseases with varying imaging features. Thus, differential diagnosis through imaging is limited [25,26]. Development and validation of a fast and noninvasive method equal to histologic results is urgently needed. Here, we developed a cancerous region classifier using a deep learning network with true labels from radiology-pathology registration. The FCN-101 model was superior to the DeepLabV3 model in terms of PA (86.91% vs 69.55%; *P*=.002) and DSC (77.47% vs 69.90%; *P*<.001). Recall values were 54.64% and 58.46%, with no significant difference observed between them (*P*=.80). The FCN-101 model excelled in identifying cancerous regions (5,846,319 vs 4,649,445 pixels; *P*<.05), whereas DeepLabV3 was more accurate for normal tissue (2,440,670 vs 1,895,664 pixels; *P*<.05) in pixel-level predictions. In the clinical workflow, the model could segment cancerous regions in grayscale ultrasound images of the breast. The results highlight the model’s potential for advancing breast cancer assessment at the microscopic level via ultrasound imaging.

Efforts have been made to establish a methodology pertaining to image-histopathology registration [27-32]. For example, Ward et al [27] and Kwak et al [28] demonstrated accurate alignment between MRIs and digital histopathologic analyses in patients with prostate cancer. In addition, Wildeboer et al [29] established a multiparametric machine learning on ultrasound for histopathology localization of prostate cancer. However, similar research in breast imaging remains limited. In our study, WSIs from CNB provided accessible clinical data suitable for state-of-the-art deep learning algorithms.

Thus, a strength of this study is that we combined accessible CNB tissue samples with real-time ultrasound imaging to facilitate the registration process. The biopsy needle targeted the desired section plane while the tissue sample was obtained at the same level, which was crucial for registration. Moreover, HFUS offers spatial tissue distribution data, enhancing dynamic biopsy procedures and clinical utility. However, subtle registration errors existed; for example, the biopsy needle was deflected when encountering small breast tumors or rigid glandular tissue [33]. To address this, our skilled operators actively mitigated needle deflections. Pre-fire and post-fire ultrasound images were compared to ensure that the extracted tissue precisely corresponded to the visualized ultrasound plane.

Studies have investigated the biopsy efficacy between existing methods. Currently, three image-guided breast biopsy techniques are used: stereotactic-, ultrasound-, and MRI-guided biopsies. Stereotactic biopsy is indicated for calcifications, and MRI-guided biopsy is indicated for lesions visible only on MRI; both primarily assess ductal proliferative lesions. Ultrasound-guided biopsy, however, applies to a broader range of breast lesions [34]. Yashima et al [35] retrospectively compared the positive biopsy rate in 453 patients with 500 lesions that underwent ultrasound-guided core needle biopsy or vacuum-assisted biopsy and reported positive biopsy rates of 61.9% and 72.4% (*P*=.032), respectively. Unrepresentative CNB specimens might not fully reflect the overall characteristics of the tumor [17]. Although multipoint sampling and repetitive biopsy increased the detection rate, they also increased complications like bleeding, infection, and tumor spreading [36]. Based on this, our model may generate real-time cancer prediction heatmaps, where suspicious regions are highlighted to assist biopsy site selection during CNB sampling. However, prior to clinical use, the proposed approach should undergo rigorous supervision and ethical evaluation to ensure its safety and reliability in guiding biopsy decisions.

The visualized cancerous map was used to enhance the interpretability of the model. Here, we found that distribution maps facilitated the assessment of cancerous regions by highlighting the hypoechoic area in HFUS images (illustrated in Figure 5), which is in accordance with clinical routine. Furthermore, areas of abnormal echogenicity or edges of lesions often corresponded to cancerous regions identified in histopathology, which was also correctly predicted by the deep learning models. Guided by the ultrasound-based CNN algorithm, doctors could identify suspicious regions based on predicted cancerous regions, even in small lesions, helping to ensure sufficient and representative CNB samples for accurate histopathological assessment. However, the model is intended to assist clinicians and cannot replace their judgment in biopsy decision-making.

Notably, there is a discrepancy between the per-image recall and the pixel-level aggregated recall, which can be explained by the scale-dependent nature of these two evaluation strategies. This scale-dependent discrepancy indicates that the current model achieves higher sensitivity for larger tumors, while the detection of small lesions remains more challenging. It highlights an important direction for future optimization aimed at improving detection stability across different tumor sizes. Although the two models had advantages in predicting cancerous regions, the confusion matrix analysis revealed a relatively low specificity, particularly for the FCN-101 model (43.07%). This indicates the presence of false-positive segmentation, with a proportion of normal background pixels being incorrectly segmented as cancerous. Such oversegmentation may be acceptable in identifying suspicious regions when emphasizing sensitivity. However, in the context of biopsy guidance, such behavior highlights the need for further optimization toward more precise boundary discrimination. Future work will focus on improving specificity through better loss function design, boundary-aware learning, and postprocessing strategies.

### Limitations

This study combined registered WSIs and HFUS images to enhance cancerous region recognition in breast cancer, which has not been well-established in the literature. Still, we acknowledge the limitations of this study. First, it is a single-center prospective study with a small and potentially homogenous dataset, which undermines the model’s generalizability and heightens the risk of overfitting. Besides, the small sample size in the test set limits statistical power and the generalizability of the prospective findings. To address this, we plan to incorporate data from multiple centers for robust external validation and conduct prospective studies to explore the models’ role in assisting breast biopsy and postoperative follow-up after neoadjuvant therapy. Second, the relatively low recall indicates that some malignant regions may be missed; future studies will focus on improving model sensitivity through ensemble and data balancing approaches. Besides, benchmarking against widely adopted baseline models (eg, U-Net) could be performed in future research. Third, there is an absence of molecular subtype or pathological classification analysis and an imbalance of specific subtypes with small sample sizes (ductal carcinoma in situ in the test set). Given that different subtypes and cancer types exhibit distinct morphological features, a subtype-specific analysis could reveal performance differences and lead to refined models optimized for specific subtypes. Fourth, other imaging modalities such as color Doppler flow imaging, elastography, and contrast-enhanced ultrasound also play paramount roles in breast cancer diagnosis. For example, the lower prevalence of internal vascularity in the test set compared with the training set may have influenced model performance. Combining the information from multiple modalities could potentially further improve the performance of the CNN model. Fifth, the ultrasound-histopathology registration was based on biopsy WSIs and ultrasound images of the needle tract. Spatial correspondence should be regarded as approximate, and pixel-level metrics may overestimate the physical precision, which necessitates further refinement and validation via the whole-mount specimen.

### Conclusion

In conclusion, we have proposed and evaluated deep learning models to identify cancerous regions in breast cancer in HFUS images through spatial registration of breast biopsy WSIs and HFUS images. This technique is potentially useful in conventional ultrasound examinations and ultrasound-guided breast biopsy procedures.

## Supplementary material

10.2196/81181 Multimedia Appendix 1 Supplementary figures and notes illustrating the modeling framework and statistical evaluation methodologies used in this study.

## Abbreviations

BI-RADS: Breast Imaging Reporting and Data System
CNB: core needle biopsy
CNN: convolutional neural network
DSC: Dice similarity coefficient
H&E: hematoxylin and eosin
HFUS: high-frequency ultrasound
MRI: magnetic resonance image/imaging
PA: pixel accuracy
ROI: region of interest
WSI: whole slide image
