# Supplementary material for: Identification and Localization of Breast Tumor Components via a Convolutional Neural Network Based on High-Frequency Ultrasound Combined With Histopathologic Registration: Prospective Study
Source: JMIR Med Inform. 2026 Jan 23;14:e81181. doi: 10.2196/81181 (PMC12829891; doi:10.2196/81181)
Supplement: Multimedia Appendix 1 [file medinform-v14-e81181-s001.docx]

**Supplementary Figures
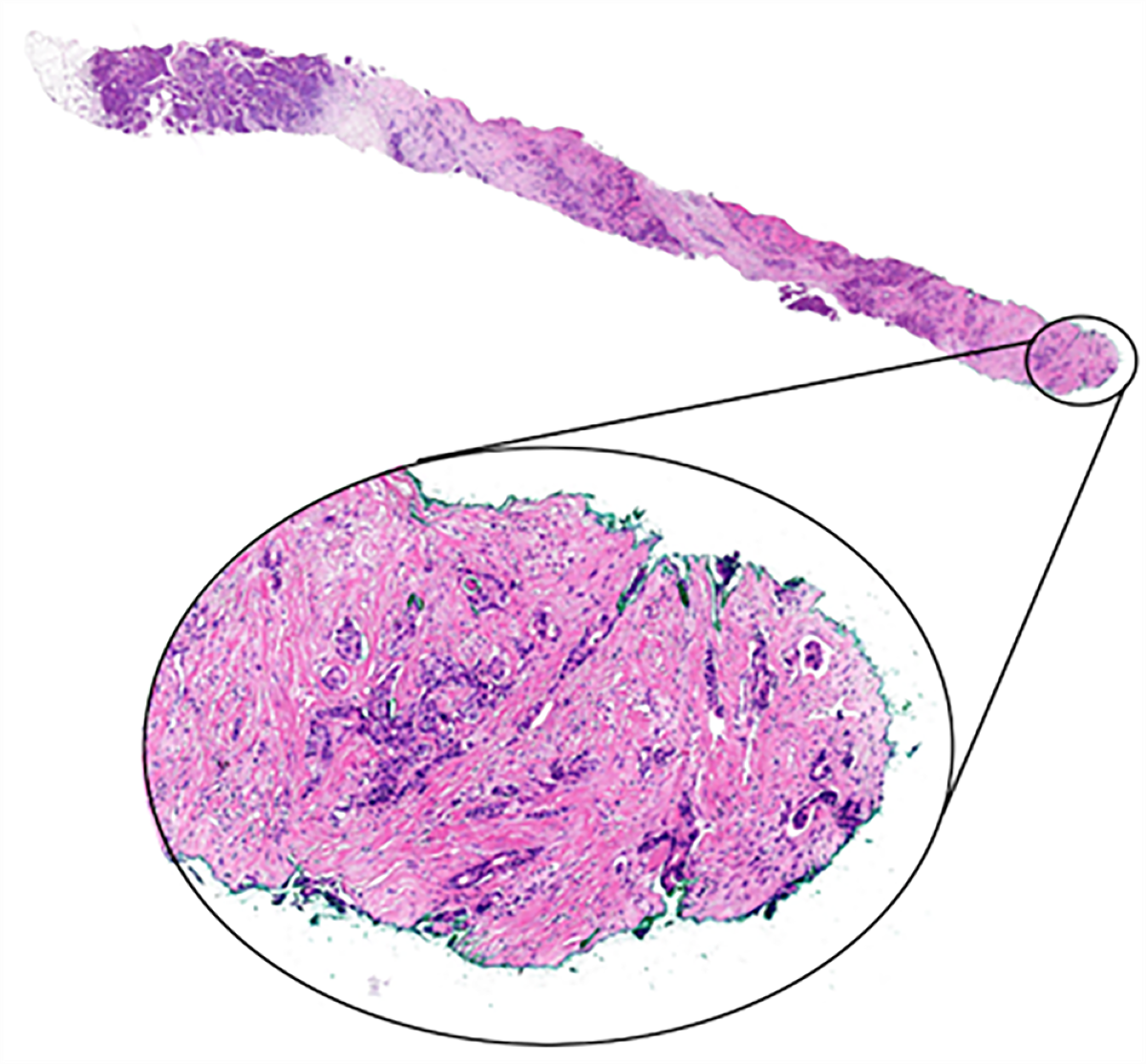
**

**Figure S1.** The needle tip side of tissue was stained for registration.

**
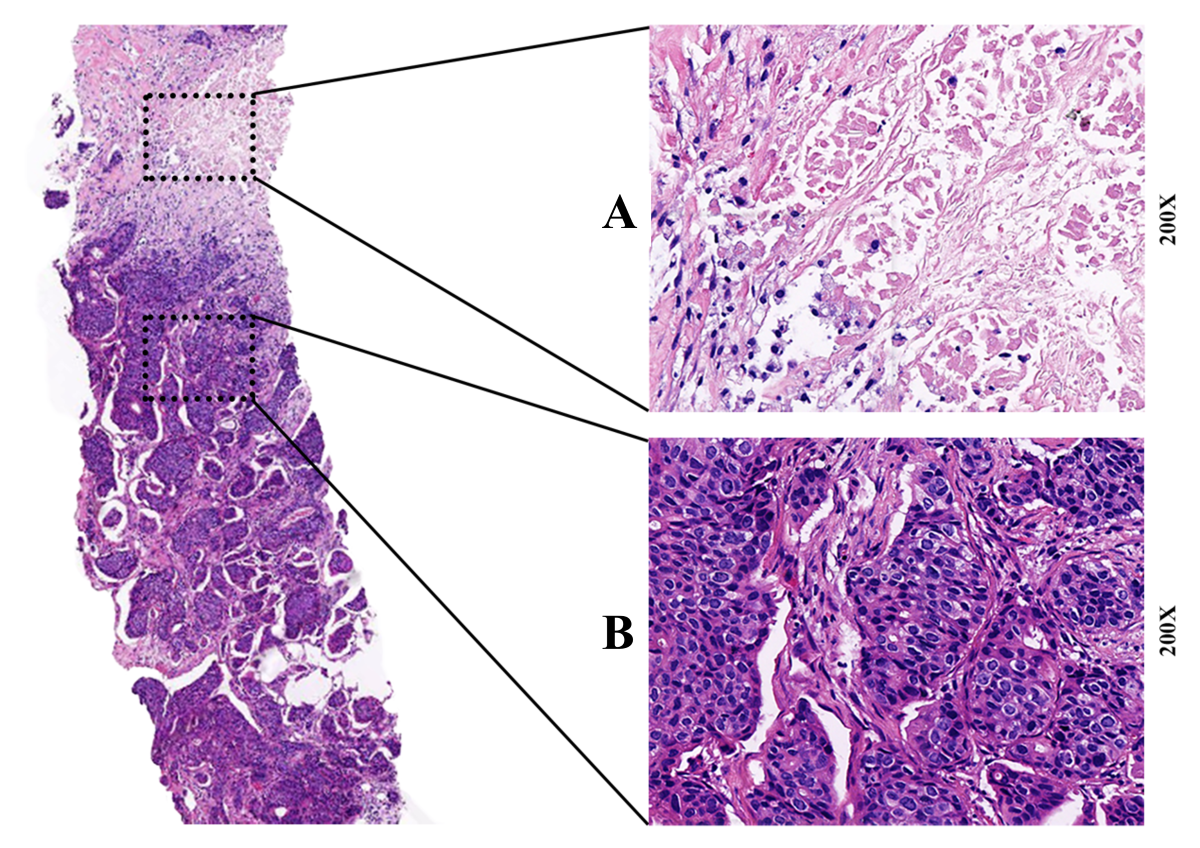
**

**Figure S2.** Representative images for histopathologic evaluation of WSIs. Highly heterogeneous breast cancer in the tissue specimen was separated into an interstitial area (A) and a cancer cell area (B) after×200 magnification.


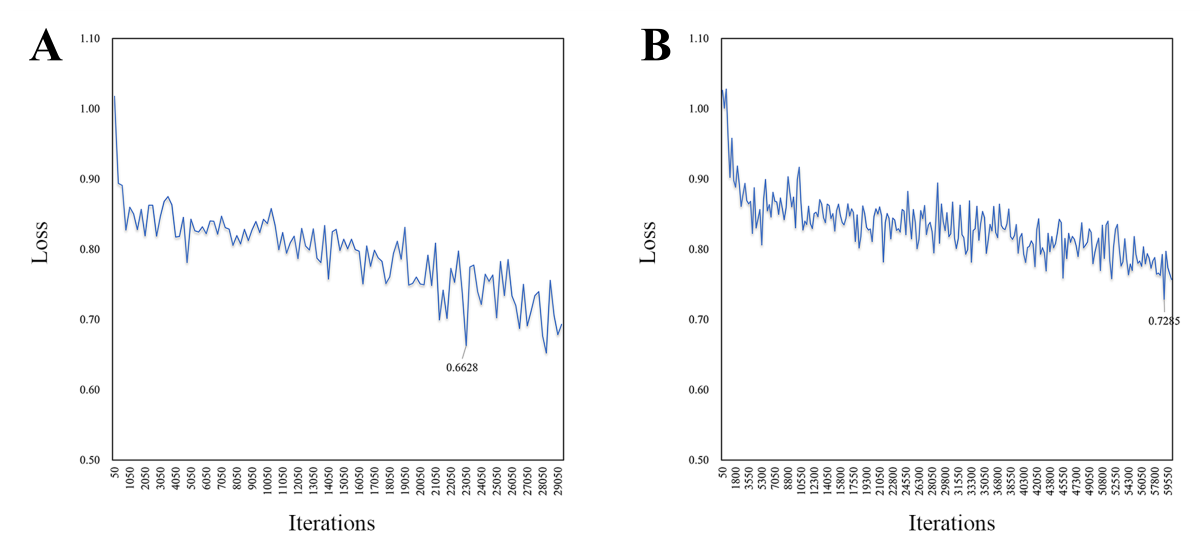


**Figure S3.** Supervision of loss function and learning process of FCN-101 (A) and DeepLabV3 (B). The loss function indicated an appropriate learning rate and suitable fitting.

**
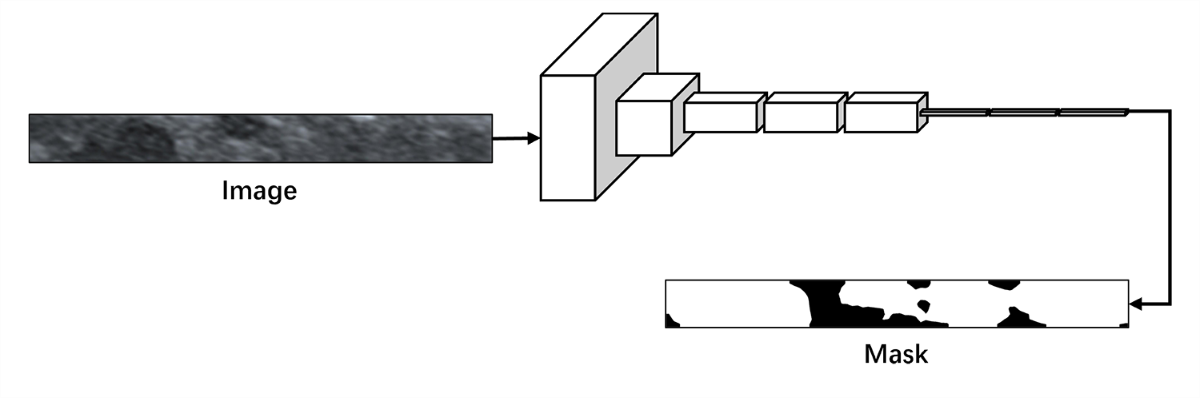
**

**Figure S4.** Basic architecture of FCN-101 model. FCN: fully convolutional network.

**
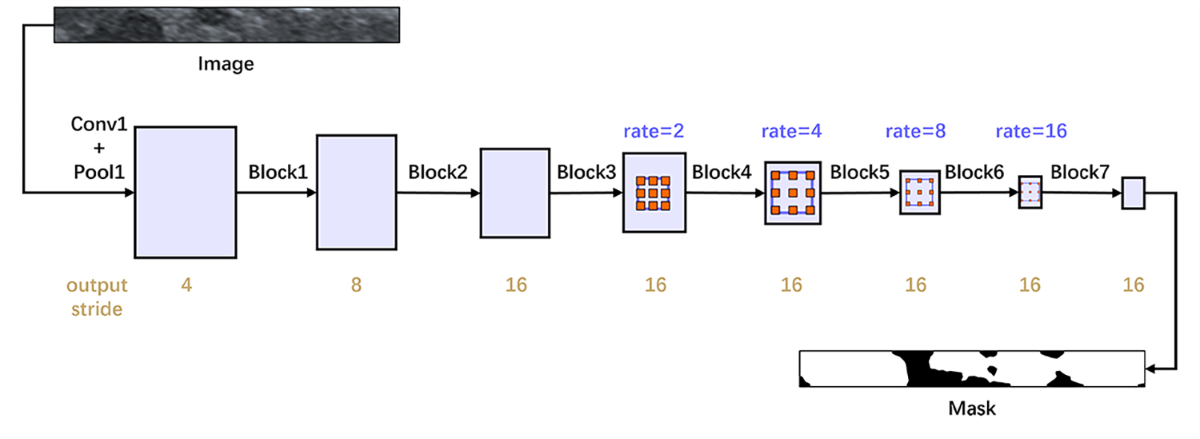
**

**Figure S5.** Basic architecture of DeepLabV3 model.

**Supplementary Methods**

Convolutional Neural Network Model Establishment

***FCN-10******1 Model—***

Fully convolutional network (FCN), a groundbreaking method for semantic segmentation, performs segmentation tasks by pixel-level classification (**Figure S4**). Distinct from the traditional convolutional neural network (CNN) model, FCN-101 replaces fully connected layers with convolutional layers [21]. The pooling layers are then used to downsample the image, reducing spatial resolution and compressing data information. This process creates feature maps in different scales, which are further upsampled back to the original image size through transposed convolution. This process ensures that every pixel is classified while maintaining both global context and local details, which is crucial for accurate semantic segmentation in medical imaging.

The key component in the FCN-101 model is transposed convolution. It upsamples the compressed feature maps gradually. The FCN-101 model further employs skip connections, which integrate feature maps from intermediate layers into the upsampling process.

To identify and localize cancerous regions in breast cancer in ultrasound images of needle tract, difficulties exist in that tumor boundaries are unclear and fewer differences in color and texture are observed compared to surrounding tissues. Traditional pixel classification methods based on fully connected layers cannot effectively capture these subtle details. In this regard, FCN-101 retains spatial details and regains high resolution through transposed convolution, which enables outlining cancerous pixels precisely.

***DeepLabV3 Model—***

DeepLabV3 is an exemplary deep learning model in semantic segmentation tasks with ResNet as the backbone [22]. It introduces an atrous spatial pyramid pooling (ASPP) module and a multi-scale handling mechanism to address the trade-off between receptive field and resolution when processing high-resolution images (**Figure S5**).

The ASPP module captures more contextual information by using parallel dilated convolutions with varying dilation rates. Additionally, ASPP applies multi-scale processing to handle tumors in different sizes. Finally, it combines global average pooling to extract local features to supplement global semantic information.

This fusion of multi-scale context information allows the model to capture both global features and local details, which is crucial for accurate segmentation in medical images where tumor sizes and shapes can vary significantly.

***Training the CNN model***

The CNN model was specifically designed to segment cancerous regions in breast cancer in high-frequency ultrasound (HFUS) images. It was trained on 386 ultrasound images of needle tract, with biopsy whole slide images (WSIs) as reference. The Python deep-learning framework was run on a system equipped with GeForce RTX 3090 GPUs. (Environmental setting: python = 3.7.1, torch = 1.11.0+cu113, torchvision = 0.12.0+cu113 and torchaudio = 0.11.0). The ResNet-34 pre-trained model weights were used as the foundation for feature extraction to learn general features. These pre-trained features are transferred to the specific task of tumor segmentation, serving as the input of the next stage of CNN model to improve capacities and reduce computation load.

In the training phase, a batch-based, iterative training strategy was deployed. This approach helped reduce variance during parameter updates, leading to more stable convergence and also allowing for efficient gradient computation through matrix operations. To ensure equity, the gross iteration was set to 25000. The model was validated every 2,500 iterations and saved every 5,000 iterations. Key metrics such as loss values, accuracy, and mean Intersection over Union are continuously monitored during training to ensure the model converges stably and avoid overfitting.

Cross-entropy loss function and Dice loss function were combined to address the imbalance in medical imaging where tumor regions are often small, whilst background occupies larger area. Cross-entropy loss function accelerates the training process by providing a robust gradient for updating model parameters. On the other hand, the Dice loss function captures the similarity between the segmentation results and ground truth, which is helpful for small cancer with unclear borders.

To prevent overfitting, several regularization techniques were introduced, including weight decay and L2 regularization. These methods are adopted to limit the model's complexity and prevent overfitting when applied to a small dataset. Additionally, learning rate schedulers and early stopping strategies were implemented to halt the training process automatically when the model's performance no longer improves. Metrics such as pixel accuracy and mean Intersection over Union were monitored continuously to evaluate the model's performance, ensuring that it achieves optimal results on the validation set during iterations.

Evaluation Metrics

To objectively and comprehensively evaluate the accuracy and similarity of the predictions made by the model's automatic segmentation on a per-image basis, the following metrics are used: pixel accuracy, dice similarity coefficient, mean Intersection over Union, precision, and recall. The definitions and calculation formulas for each metric are as follows:

***Pixel Accuracy***

Pixel accuracy (PA) is the proportion of correctly segmented pixels to the total number of pixels in an image. It is defined by the following formula:

$$\begin{aligned} PA=\frac{\sum_{i=0}^{k} p_{ii}}{\sum_{i=0}^{k} \sum_{j=0}^{k} p_{ij}} \end{aligned}$$

where $p_{ii}$ represents the total number of pixels where both the actual and predicted categories are *i*, and $p_{ij}$ represents the total number of pixels where the actual category is *i* and the predicted category is *j.*

***Dice Similarity Coefficient***

Dice similarity coefficient (Dice), also known as the overlap index, is a set similarity measure used to evaluate the similarity between the segmentation result and the ground truth. It is defined by the following formula:

$$\begin{aligned} Dice=\frac{2\left| A\cap B \right|}{\left| A \right|+\left| B \right|} \end{aligned}$$

where *A* and *B* represent the ground truth and the predicted segmentation image, respectively.

***Mean Intersection over Union***

Mean intersection over union (mIoU) is the average ratio of the intersection to the union between the predicted results and the ground truth for each category. It is defined by the following formula:

$$\begin{aligned} mIoU=\frac{1}{k+1}\sum_{i=0}^{k} \frac{p_{ii}}{\sum_{j=0}^{k} p_{ij}+\sum_{j=0}^{k} p_{ji}-p_{ii}} \end{aligned}$$

where $p_{ii}$​ represents the total number of pixels where both the actual and predicted categories are *i*, and $p_{ij}$​ represents the total number of pixels where the actual category is *i* and the predicted category is *j*.

***Precision***

Precision (*PRE*), also known as positive predictive value, is the proportion of correctly classified pixels among the total number of pixels predicted as a particular category. It is defined by the following formula:

$$\begin{aligned} PRE=\frac{TP}{TP+FP} \end{aligned}$$

where True positive (*TP*) refers to the number of pixels where the model's predicted category matches the ground truth, and False positive (*FP*) refers to the number of pixels where the model predicts a category while the ground truth belongs to another category.

***Recall***

Recall (*REC*), also equivalent to sensitivity in instance segmentation problems, is the proportion of correctly classified pixels among the total number of true positive pixels in the ground truth. It is defined by the following formula:

$$\begin{aligned} REC=\frac{TP}{TP+FN} \end{aligned}$$

where True Positive (*TP*) refers to the number of pixels where the model's predicted category matches the ground truth, and False Negative (*FN*) refers to the number of pixels where the model predicts a different category while the ground truth belongs to the predicted category.

**Reference:**

21. Dung CV, Anh LD. Autonomous concrete crack detection using deep fully convolutional neural network, AUTOMATION IN CONSTRUCTION. Mar 2019;99:52-58. [doi: 10.1016/j.autcon.2018.11.028]

22. Chen LC, Papandreou G, Schroff F, et al. Rethinking atrous convolution for semantic image segmentation. arXiv. 2017;1706.05587. [doi.org/10.48550/arXiv.1706.05587]
